# Quantification of intrinsic subtype ambiguity in Luminal A breast cancer and its relationship to clinical outcomes

**DOI:** 10.1186/s12885-019-5392-z

**Published:** 2019-03-08

**Authors:** Neeraj Kumar, Dan Zhao, Dulal Bhaumik, Amit Sethi, Peter H. Gann

**Affiliations:** 1 Department of Pathology, College of Medicine, University of Illinois at Chicago, 840 S. Wood Street, MC 847, Chicago, IL 60612 USA; 2 Division of Epidemiology and Biostatistics, School of Public Health, University of Illlinois at Chicago, 1603 W. Taylor Street, MC 923, Chicago, IL 60612 USA; 3 Department of Electrical Engineering, Indian Institute of Technology Bombay, Room 200, Mumbai, PIN-400076 India

**Keywords:** Intrinsic subtype, Intratumor heterogeneity, PAM50, Luminal a

## Abstract

**Background:**

PAM50 gene profiling assigns each cancer to a single intrinsic subtype. However, individual cancers vary in their adherence to a prototype, and due to bulk tissue sampling, some may exhibit expression patterns that indicate intra-tumor admixture of multiple subtypes. Our objective was to develop admixture metrics from PAM50 gene expression profiles in order to stratify Luminal A (LumA) cases according to their degree of subtype admixture, and then relate such admixture to clinical and molecular variables.

**Methods:**

We re-constructed scaled, normalized PAM50 profiles for 1980 cases (674 LumA) in the METABRIC cohort and for each case computed its Mahalanobis (M-) distance from its assigned centroid and M-distance from all other centroids. We used t-SNE plots to visualize overlaps in subtype clustering. With Normal-like cases excluded, we developed two metrics: Median Distance Criteria (MDC) classified pure cases as those located within the 50th percentile of the LumA centroid and > =50th percentile from any other centroid. Distance Ratio Criteria (DRC) was computed as the ratio of M-distances from the LumA centroid to the nearest non-assigned centroid. Pure and admixed LumA cases were compared on clinical/molecular traits. TCGA LumA cases (*n* = 509) provided independent validation.

**Results:**

Compared to pure cases in METABRIC, admixed ones had older age at diagnosis, larger tumor size, and higher grade and stage. These associations were stronger for the DRC metric compared to MDC. Admixed cases were associated with HER2 gain, high proliferation, higher PAM50 recurrence scores, more frequent *TP53* mutation, and less frequent *PIK3CA* mutation. Similar results were observed in the TCGA validation cohort, which also showed a positive association between admixture and number of clonal populations estimated by PyClone. LumA-LumB confusion predominated, but other combinations were also present. Degree of admixture was associated with overall survival in both cohorts, as was disease-free survival in TCGA, independent of age, grade and stage (HR = 2.85, Tertile 3 vs.1).

**Conclusions:**

Luminal A breast cancers subgrouped based on PAM50 subtype purity support the hypothesis that admixed cases have worse clinical features and survival. Future analyses will explore more extensive genomic metrics for admixture and their spatial significance within a single tumor.

**Electronic supplementary material:**

The online version of this article (10.1186/s12885-019-5392-z) contains supplementary material, which is available to authorized users.

## Background

Intra-tumor heterogeneity, due to genomic instability and the emergence of subclones in various regions of a tumor, challenges our ability to predict outcomes or response to targeted therapy for individual patients. It is therefore a major obstacle to the further development of precision oncology for women with breast cancer [1–3]. Current breast cancer classification, by immunohistochemistry (IHC) or intrinsic subtyping by RNA profiling, focuses on inter-tumor heterogeneity. It assumes that each patient belongs to a single discrete class, even though variation within these subtypes is acknowledged and evidence suggests that subtype admixture is relatively common [4–9]. At present, intra-tumor heterogeneity is incompletely understood and even more incompletely quantified [10–12]. Multi-region sampling by DNA sequencing, including single-cell sequencing, can reveal molecular heterogeneity in rich detail; however, these and other genomic techniques are costly and poorly suited for mapping spatial variation within a tumor. Given the ubiquity of intra-tumor variation, it is recognized that bulk sampling of tumors, as is typical for genomic analysis, inadvertently captures a portion of this diversity [1, 13]. Accordingly, a number of computational methods have been developed to infer and measure intra-tumor heterogeneity based on genomic data from single-region bulk samples [14–16]. In contrast to these important efforts at characterizing *global* genomic heterogeneity, our approach, by identifying cases that are ambiguous with respect to a class prototype, focuses on *subtype heterogeneity* and the potential coexistence of multiple subtypes within a single breast cancer. Our underlying premise is that intrinsic subtypes represent favored pathways for clonal evolution in breast cancer, and that a straighforward metric based on adherence to an assigned subtype versus adherence to an alternate subtype is a reflection of subtype admixture.

The primary aim of this study was to develop quantitative metrics for evaluating subtype purity in breast cancers designated as Luminal A by the PAM50 gene expression profile. PAM50 classification has consistently shown the capacity to add information to clinical variables regarding prognosis and treatment response in hormone receptor positive breast cancers [17, 18]. However, the importance of subclonal diversity in luminal breast cancers is implicit in the ASCO/CAP criteria for ER positivity by immunohistochemistry, which require only that greater than 1% of the cells stain positive for ER, thus implying that other regions might not have a luminal phenotype [19]. Lower percentages of ER and PR staining, in fact, are associated with shorter recurrence-free survival in hormone receptor positive breast cancer [20]. We initially focus on Luminal A cases because admixture with any of the other major intrinsic subtypes (Luminal B, HER2-enriched, Basal) would be linked, hypothetically, to more aggressive disease and worse outcomes.

In this study, we used PAM50 gene expression profiles among Luminal A cases in the METABRIC and TCGA cohorts to generate two novel measures of subtype admixture, and then used those to test the hypothesis that ambiguous and potentially admixed cases have more adverse tumor characteristics and worse survival than pure ones.

## Methods

### Study populations and data sources

We developed and validated the proposed heterogeneity quantification approach using mRNA expression data from two independent, publicly available breast cancer cohorts – the Molecular Taxonomy of Breast Cancer International Consortium (METABRIC) cohort containing 17,814 unique genes from 1980 patients, and The Cancer Genome Atlas (TCGA) BRCA provisional cohort containing 20,532 unique genes from 1081 patients. In METABRIC, mRNA was measured with the Illumina HT-12v3 platform and CNA with the Affymetrix SNP 6.0 array. TCGA mRNA data was obtained using the Illumina HiSeq platform. All clinical and genomic data for the METABRIC and TCGA cohorts were downloaded from cBioportal for Cancer Genomics (http://cbioportal.org). The METABRIC data was previously median-centered and log-transformed; any missing, zero or negative gene expression values in METABRIC were replaced by real numbers randomly sampled from a uniform distribution in the range [.05,.95]. We applied the log_2_(X + 1) transformation for the centered TCGA data before analysis. METABRIC and TCGA datasets were used for model development and independent validation, respectively. The Office for the Protection of Research Subjects at the University of Illinois at Chicago reviewed and approved use of the data.

Gene expression profiles of PAM50 genes from both METABRIC and TCGA datasets were extracted from the pre-processed data. While all 50 PAM50 genes are available in the TCGA dataset, only 47 genes were available in METABRIC. For both METABRIC and TCGA datasets, previously reported algorithms for intrinsic subtype calls were used to assign each case to one of five PAM50 subtypes (Luminal A, Luminal B, HER2, Basal and Normal) [21]. Zhao, et al. demonstrated that the accuracy of PAM50 classification can be affected by extreme differences in the prevalence of ER-positive cases between a study cohort and the benchmark training cohort [22]. However, their simulation results indicate acceptably low error rates throughout an ER-positive prevalence range of about 60–80%, and this prevalence was 75.6 and 73.5% in METABRIC and TCGA, respectively. Our re-computed PAM50 classifications were identical to those recorded in the downloaded datasets. Claudin-low cases were identified using the labels assigned in the METABRIC database, which were based on the gene expression profile predictor from Prat, et al. [23]. Claudin-low labels were not available in TCGA.

### Data representation

We computed centroids of the PAM50 gene expression profile for each of the six subtypes. We then computed the Mahalanobis distance (M-distance) between the PAM50 gene expression profiles of each case with each of the six prototypical centroids using the covariance matrices of the respective subtype clusters [24]. We used M-distance instead of Euclidean distance because the former can account for different covariance among the PAM50 gene subtype clusters. We created two-dimensional t-distributed Stochastic Neighbor Embedding (t-SNE) plots to visualize the clustering patterns of different subtype classes [25].

### Subtype admixture metrics

We developed two metrics to quantify genomic heterogeneity of Luminal A tumors. The small number of cases classified as Normal were excluded from admixture quantification since discriminating these cases from benign tissue is uncertain and, in any event, admixture of Luminal A with this subtype would not be expected to worsen outcomes. For the first metric, we created a categorical variable determined by the M-distance rank of a case to its assigned centroid and its rank for the most likely alternative centroid. A case was classified as ‘Pure’ if it was located within a specified rank threshold of its assigned subtype while not being located within that threshold distance for any other subtype. Cases were considered ‘Admixed’ if they were situated beyond the threshold for the assigned class while within the threshold for any alternative class. Finally, cases that were beyond the threshold for both the assigned and alternate classes were designated as ‘Neither’ as these were neither Pure nor Admixed. Cases that were near both the assigned centroid and an alternate centroid (hence were neither Pure nor Admixed) were non-existent for the thresholds that were found to be relevant for this analysis. To determine optimal threshold distances, we used three-fold cross-validation to compare various incremental M-distance threshold values according to the difference in overall survival between Pure vs. Admixed Luminal A cases, based on log-rank *p*-value. Graphing showed that *p*-values plateaued near the 50th percentile, with a minimum for Luminal A cases in the METABRIC cohort at the 49th percentile. We selected the 50th percentile as M-distance threshold for simplicity. Hence, we termed this method of admixture identification as the Median Distance Criteria (MDC).

The optimal distance threshold was re-computed for the TCGA cohort, since differences in the data distribution affect the relationship between prototypical centroids for each PAM50 class, and it was found to be 31st instead of the 50th percentile. Since the optimal selection of M-distance threshold depends on the gene expression and survival distribution for an individual cohort, we consider this categorical type of admixture metric to be suitable only for proof-of-principle and not for validation of a fixed algorithm. Therefore, we also developed a second continuous rather than discrete metric, based on the ratio of M-distances for assigned versus alternate subtypes.

### Distance ratio criteria

For each Luminal A case in METABRIC cohort, we first identified the nearest non-assigned centroid among the Luminal B, Basal, HER2 and Claudin-low centroids. Each Luminal A case was then assigned a score given by the ratio of its M-distance from the Luminal A centroid to its M-distance to the nearest non-assigned centroid. We stratified Luminal A cases by tertiles of this ratio and call this method Distance Ratio Criteria (DRC) for admixture quantification. DRC was also applied to Luminal A cases in the TCGA cohort for an independent validation. We denote the first, second, and third tertiles by T1, T2 and T3, respectively. T1 contained the purest (homogeneous) Luminal A cases, while T3 contained maximally admixed ones. Cases far from all centroids tended to have intermediate scores and thus were predominantly in T2. We selected the 10 purest and 10 most admixed cases of each major subtype, based on DRC, and used hierarchical clustering (Euclidean distance, average linkage) of PAM50 gene expression to visualize the relationship of purity to clustering patterns and identify the genes driving those patterns.

### Associations with clinical features, molecular characteristics and survival

To test the hypothesis that heterogeneous Luminal A cases are more likely to have adverse clinical features at diagnosis than homogeneous ones, we compared the two extreme sub-classes of Luminal A cases as determined by MDC – Pure and Admixed. Variables compared included mean age at diagnosis, menopausal status, percentage that were clinically ER+, PR+, and HER2+, percentage that were clinical Luminal A as defined by a surrogate definition (ER+ or PR+, HER2- and low-proliferation by AURKA expression), tumor size, Nottingham grade, percentage with positive nodal status, and percentage with stage greater than I. We performed similar analyses to compare T1 and T3 sub-classes of Luminal A cases as determined by DRC.

We also compared pure versus admixed Luminal A cases on molecular characteristics, including HER2 gain determined by HER2 SNP6 DNA microarray, proliferation status determined by AURKA expression [26, 27], PAM50 based 11-gene Proliferation score [28], PAM50 based risk of recurrence (ROR) score [21], mutational load, and mutant allele tumor heterogeneity (MATH) score [29]. For TCGA cases we ran PyClone to determine the number of clonal populations in each Luminal A case [30]. MATH estimates the degree of intra-tumor genetic heterogeneity based on simple variant allele frequencies, whereas PyClone uses Bayesian modeling to detect distinct cell populations based on clustering of somatic mutations, while accounting for copy number alterations. We used two sided t-tests and chi-square statistics to compare means and percent of cases between purity groups as determined by MDC and DRC. We also ranked and compared the prevalence of somatic mutations within purity sub-classes by Chi-square *P* values to determine which mutations were most strongly associated with admixture. In METABRIC, we compared overall survival for Luminal A cases using Kaplan-Meier curves as well as hazard ratios from unadjusted and adjusted Cox proportional hazards models. Factors for adjustment included age, tumor grade, stage, and tumor size. We performed similar analyses in TCGA for both overall and disease-free survival, except that data on tumor grade was not available for covariate adjustment.

## Results

### Visualizing the pair-wise low-dimensional embedding of METABRIC luminal a patients

**Fig. 1:**
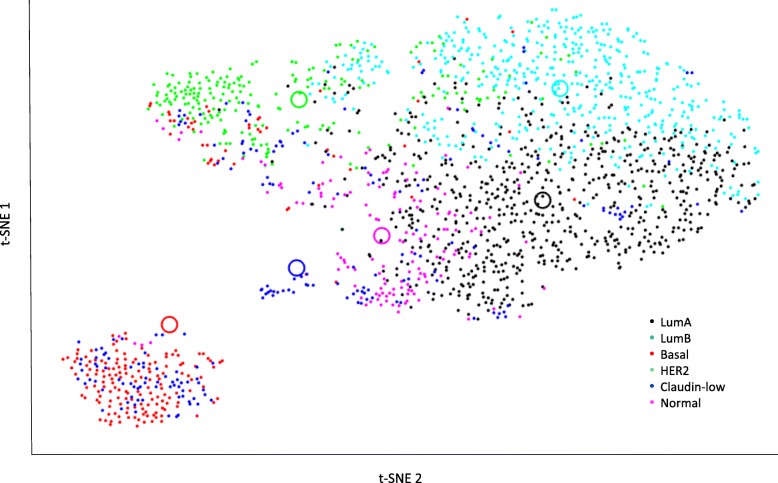
t-SNE plots for all subtypes in METABRIC cohort, showing: **1**) luminal subtypes cluster relatively closely, with basal cluster highly distant, **2**) substantial admixture across subtypes as indicated by cases located near non-assigned centroids. (t-SNE centroids = open circles)

Of the 1980 cases in the original METABRIC cohort, 1888 had adequate mRNA data for analysis. Figure 1 shows a two-dimensional visualization of PAM50 gene expression profile clustering using t-SNE software. Significant overlap is evident between various PAM50 clusters, with cases assigned to the Basal sub-type forming the only visually distinct cluster.

**Fig. 2:**
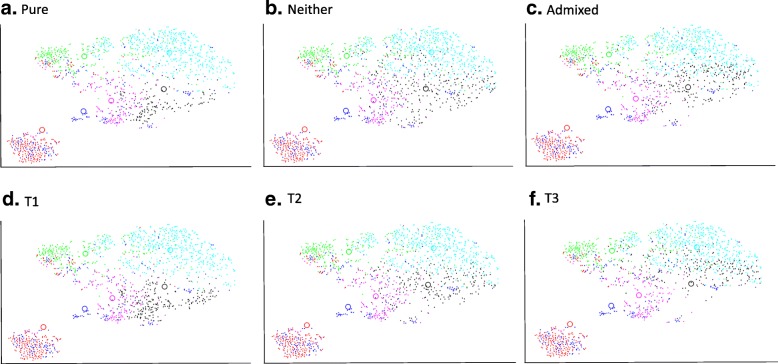
Subtype clustering shown by t-SNE plots for each purity category in the METABRIC cohort. Luminal A cases (black dots) migrate towards other regions and predominant co-clustering of Luminal A with Luminal B cases (cyan) is evident as admixture increases. **a**-**c**: Median Distance Criteria; **d**-**f**: Distance Ratio Criteria tertiles. Other subtype colors: Basal (red), HER2 (green), Claudin-low (blue), Normal (fuchsia)

**Fig. 3:**
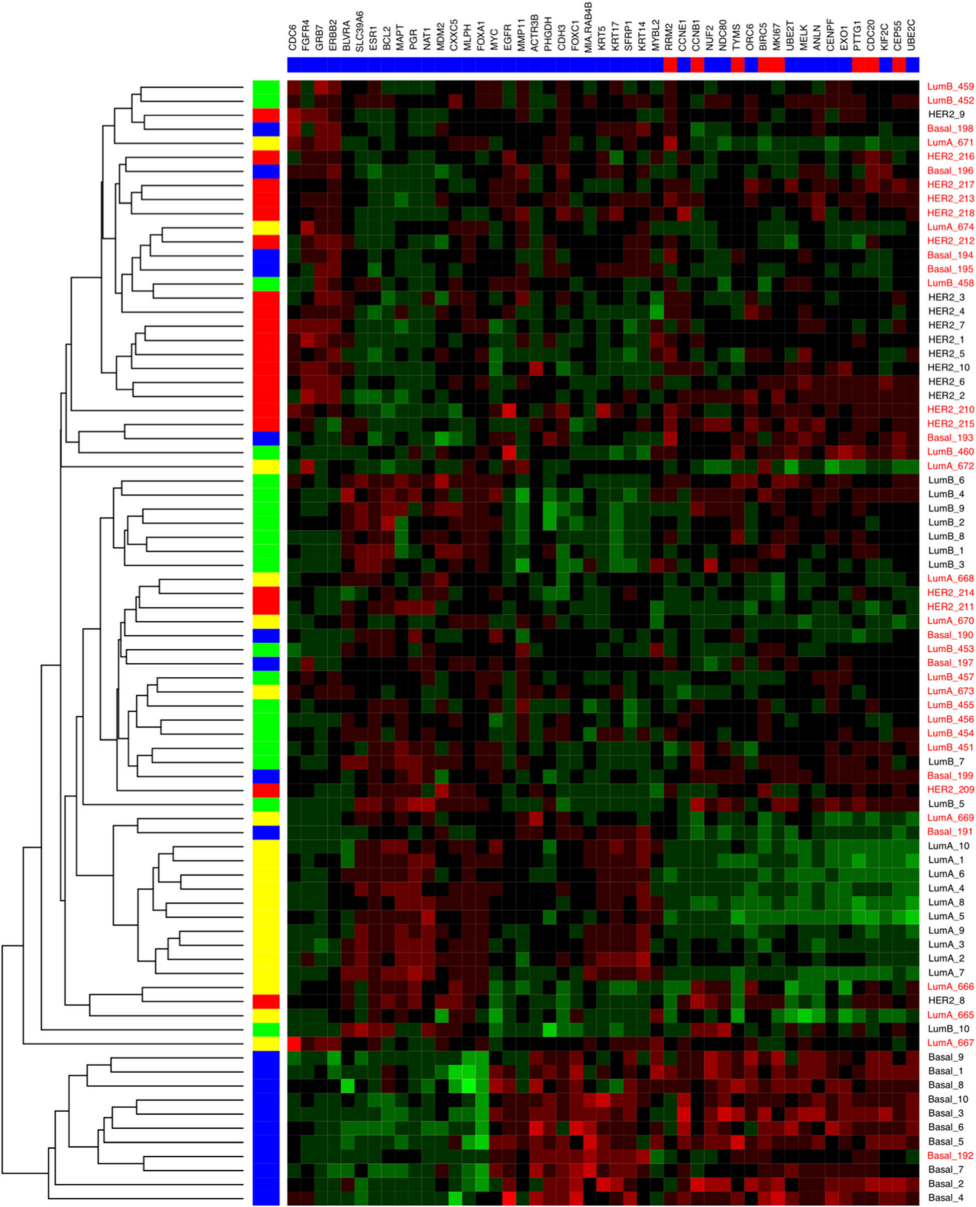
PAM50 gene hierarchical clustering of the ten purest and ten most admixed cases (based on DRC) for each major subtype in METABRIC. Vertical bar colors show assigned PAM50 subtype. Blue = Basal, Red = HER2, Yellow = LumA, Green = LumB. Far right column shows DRC rank within assigned subtype: pure in black font, admixed in red

Among the eligible cases in the METABRIC cohort, 674 (36%) were Luminal A. Figure 2 shows the t-SNE plots with distribution of PAM50 gene expression profiles of these Luminal A cases, stratified by categories per the MDC (Fig. 2a-c) and DRC (Fig. 2e-f) metrics. As expected, Pure Luminal A cases identified by MDC have the least overlap with any other PAM50 cluster and the amount of cross-cluster overlap increases as we consider Neither and Admixed sub-classes. DRC based stratification provides an even stronger evidence of overlap between the third tertile (T3 or most impure) of Luminal A cases with other PAM50 classes compared to T2 and T1 cases. T3 Luminal A cases appear most likely to overlap with Luminal B cases. Luminal B was the nearest alternate centroid for 59 and 65% of the Luminal A cases by MDC and DRC, respectively. Table 1 shows the percentages of cases with Basal, HER2, Luminal B and Claudin-low as the alternate centroid. Although PAM50 gene expression profiles of Normal cases were not used in quantifying admixture, these are shown in Figs. 1 and 2 for illustration. Figure 3 is a heat map displaying the hierarchical clustering, based on expression of individual PAM50 genes, for the 10 purest and 10 most admixed cases from each of the four major subtypes. As expected, the pure cases of each subtype cluster together; however, in agreement with the t-SNE plots, Luminal A and Luminal B cases join early in the dendrogram whereas the pure HER2 and Basal cases join late. Admixed cases of various types tend to cluster together and the admixed Luminal A cases are widely dispersed across these clusters.

**Table 1:** Nearest alternate centroids for the admixed Luminal A cases in the METABRIC cohort^a^

	*n*	percentage
Admixed by Median Distance Criteria (*n* = 243)		
Basal	2	1%
HER2	40	17%
Luminal B	144	59%
Claudin-low	57	23%
Distance Ratio tertile 3 (*n* = 229)		
Basal	2	1%
HER2	56	24%
Luminal B	148	65%
Claudin-low	23	10%

^a^Normal-like cases were excluded from admixture metric calculations

Clinical-pathological features of the 674 cases identified as Luminal A by PAM50 and their differences across Pure and Admixed sub-classes in the METABRIC cohort are shown in Table 2. The overlap in classification between PAM50 and clinical criteria for Luminal A was large but incomplete, as expected. By IHC and/or FISH, 76% were ER+, 54% were PR+, and 13% were HER2+. We created a composite variable to identify likely Luminal A cases, based on positive IHC for ER or PR, negative IHC and/or FISH for HER2, and low proliferation as indicated by AURKA gene expression. A total of 49% of the PAM50 Luminal A cases were clinical Luminal A as defined by these surrogate criteria.

**Table 2:** Clinical characteristics of patients in the METABRIC cohort with Luminal A breast cancer, classified by MEDIAN DISTANCE PURITY criteria

	Median distance criteria				
	All cases *n* = 674	Pure *n* = 143	Neither *n* = 288	Admixed *n* = 243	*P*, Pure vs Admixed
Age, years (mean)	62.8	58.9	63.3	64.5	<.001
Menopausal status (%)					
Pre-menopausal	19%	29%	16%	16%	< 0.001
Post-menopausal	81%	71%	84%	84%	–
ER+ (%)	76%	79%	75%	76%	0.533
PR+ (%)	54%	52%	53%	56%	0.459
HER2+ (%)	13%	11%	14%	13%	0.525
ER+ or PR+, HER2-, and Low proliferation^a^	49%	65%	41%	49%	0.003
Tumor size					
mm (mean)	23.8	23.3	24.3	23.6	0.775
< 20 mm (%)	36%	46%	34%	33%	0.012
> 20 mm (%)	64%	54%	66%	67%	–
Grade (mean)^b^					
score (mean)	2.08	1.85	2.13	2.14	< 0.001
< 3 (%)	71%	79%	67%	70%	0.057
= 3 (%)	25%	16%	28%	26%	–
Node positive (%)	42%	38%	40%	48%	0.056
Stage (%)^c^					
I	44%	52%	45%	39%	0.026
II-III-IV	56%	48%	55%	61%	–

^a^ ER, PR, and HER2 by IHC and/or FISH, proliferation determined by AURKA RNA expression

^b^ Nottingham score (0–3) based on nuclear appearance, mitotic cells and tubular architecture

^c^ Excluding missing data (12–21%)

A comparison of the clinical-pathological characteristics of the Pure (*n* = 143) versus Admixed (*n* = 243) sub-classes revealed several important differences. Compared to Pure cases, Admixed ones were on average 5.6 years older (*P* < 0.001) and were more likely to be post-menopausal (84% vs 71%, *P* < .001). While no statistically significant differences were observed for clinically determined ER, PR or HER2 status, more Pure cases conformed to the IHC/proliferation definition of Luminal A compared to the Admixed group (65% vs 49%, *P* = .003). Admixed tumors were also more likely to be greater than 20 mm in size than Pure ones (67% vs. 54%, *P* = 0.012) and have higher Nottingham grade (mean 2.14 vs. 1.85, *P* < 0.001). Finally, admixed tumors were more likely to be above Stage I at diagnosis (61% vs. 48%, *P* = 0.026). Tumors classified as neither Pure nor Admixed tended to have intermediate values for these clinical features.

Differences in clinical-pathological features were even more pronounced when we compared T1 (*n* = 223) and T3 (*n* = 229) cases identified using DRC, as shown in Table 3. Compared to T1 patients, T3 patients were significantly older (mean age 65.2 vs. 60.0 years, *P* < 0.001) and more likely to be post-menopausal (87% vs. 74%, *P* < 0.001). An even more substantial difference in adherence to the surrogate clinical Luminal A definition was observed between T1 and T3 cases (67% vs. 33%, *P* < 0.001) as compared to Pure and Admixed. T3 cases in comparison to T1 cases had significantly larger tumors and markedly higher Nottingham grade. T3 cases also had a higher prevalence of node positivity and stage greater than one. Overall, these results indicate that DRC is a stronger measure for identifying admixture than MDC. Despite the prevalence of more advanced disease in the T3 group, there was only a small non-significant difference in receipt of chemotherapy between T1 (6.8%) and T3 (9.4%) Luminal A cases that were clinically ER-positive. However, statistical power was weak given the small number of such cases receiving chemotherapy (*n* = 12 and 16 for T1 and T3 respectively). Surprisingly, T3 ER-positive cases were more likely than the T1 cases to have received hormone therapy (75% vs. 59%, *P* = 0.002).

**Table 3:** Clinical characteristics of patients in the METABRIC cohort with Luminal A breast cancer, classified by DISTANCE RATIO purity

	Distance ratio tertiles			
	T1 *n* = 223	T2 *n* = 222	T3 *n* = 229	*P*, T1 vs T3
Age, years (mean)	60.0	63.0	65.2	<.001
Menopausal status (%)				
Pre-menopausal	26%	17%	13%	< 0.001
Post-menopausal	74%	83%	87%	–
ER+ (%)	79%	75%	75%	0.372
PR+ (%)	54%	55%	53%	0.851
HER2+ (%)	13%	10%	16%	0.258
ER+ or PR+, HER2-, and Low proliferation	67%	49%	33%	< 0.001
Tumor size				
mm (mean)^a^	21.7	24.8	24.9	0.001
< 20 mm (%)	46%	34%	28%	< 0.001
> 20 mm (%)	54%	66%	72%	–
Grade (mean)				
score (mean)	1.86	2.05	2.32	< 0.001
< 3 (%)	81%	73%	59%	< 0.001
= 3 (%)	13%	23%	38%	–
Node positive (%)	37%	41%	49%	0.014
Stage (%)				
I	53%	42%	38%	0.006
II-III-IV	47%	58%	62%	–

^a^Linear regression with tumor size (continuous) as dependent variable: *P* = 0.006

Table 4 presents the differences in molecular characteristics for METABRIC Luminal A cases stratified by subtype purity according to DRC. T3 cases compared to T1 were more than twice as likely to have HER2 gain (17% vs 7%, *P* = 0.001), and T3 cases had substantially higher proliferation by AURKA expression, as well as higher proliferation and recurrence scores by PAM50. Mutational load, determined for 173 targeted genes sequenced in METABRIC, was not associated with degree of admixture, nor was the MATH score. The three genes whose mutation prevalence ranked highest for differentiating admixture groups were *TP53*, *PIK3CA*, and *CBFB*. Table 4 shows that *TP53* mutation, which is less common in Luminal A tumors, was in fact more common in admixed tumors, and that *PIK3CA* and *CBFB* mutations, which are associated with ER-positive tumors, were more prevalent in the purer cases. The ten top-ranked PAM50 genes differentially expressed between pure and admixed cases (T1 vs. T3) are shown in Additional file 1: Table S1. Out of the 11 proliferation-related genes in the PAM50, six were ranked among the top ten. The significant role of the proliferation genes in terms of conferring purity can also be seen in Fig. 3, in which the activity of these genes is highly concordant among pure cases and highly discordant among the admixed cases.

**Table 4:** Molecular characteristics of Luminal A breast cancers in the METABRIC cohort classified by subtype purity, *n* = 674

	Distance ratio tertiles			
	T1 *n* = 223	T2 *n* = 222	T3 *n* = 229	*P*, T1 vs T3
HER2 gain^a^ (%)	7%	9%	17%	0.001
High proliferation (AURKA)	5%	25%	41%	< 0.001
Proliferation Score, PAM50 (mean)	8.99	9.05	9.09	< 0.001
Recurrence Score, PAM50 (mean)	28.60	57.43	75.65	< 0.001
Risk of recurrence, PAM50				
Low	52%	10%	3%	< 0.001
Intermediate	38%	28%	8%	
High	10%	62%	89%	
Mutational load^b^ (mean)	5.46	5.80	5.44	0.919
MATH score^c^ (mean)	0.30	0.30	0.33	0.238
Somatic mutations (%)				
TP53	7%	10%	20%	< 0.001
PIK3CA	68%	59%	45%	< 0.001
CBFB	14%	7%	5%	0.003

^a^HER2 gain or loss determined by HER2_SNP6 DNA microarray

^b^Number of mutations among 172 selected genes sequenced

^c^MATH score = mad(vaf)/median(vaf); where mad = median absolute deviation and vaf = variant allele frequency (intratumor heterogeneity based on variation in mutant allele frequency, see Mroz EA, Oral Oncol 2013)

### Survival analysis of METABRIC luminal a patients

**Fig. 4:**
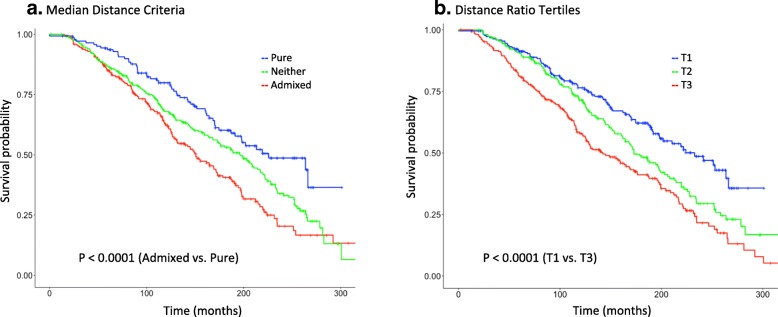
Overall survival for Luminal A breast cancer in METABRIC was significantly worse for admixed cases, determined either by Median Distance Criteria (**a**) or Distance Ratio (**b**)

Figure 4 shows the Kaplan-Meier plots of overall survival (OS) for METABRIC Luminal A cases stratified according to MDC and DRC. There was a statistically significant difference (*P* < 0.0001) between Pure and Admixed as well as T1 and T3 patients. Median overall survival time for Pure cases was 226 months compared to 151 months for the Admixed ones and was 233 months for T1 compared to 142 months for T3. The 10-year survival probability for T3 Luminal A cases was 0.58 (95% CI: 0.52–0.65), which is intermediate between the 10-year survival for all Luminal A cases (0.69; 95% CI: 0.65–0.72) and that for all Luminal B cases (0.51, 95% CI: 0.46–0.56).

Results of unadjusted and adjusted Cox-proportional hazards models for overall mortality of Luminal A cases in METABRIC stratified by the admixture metrics are presented in Table 5. In the unadjusted model, the hazard ratio for Admixed relative to Pure cases was 1.93 (95% CI: 1.42–2.63), and that for Neither to Pure was 1.48 (95% CI: 1.10–2.00). In models adjusting for age, grade, stage and tumor size, the hazard ratio for MDC Admixed cases decreased to 1.47 but remained statistically significant. Similarly, the hazard ratio for T3 to T1 cases was 1.98 (95% CI: 1.52–2.58) in the unadjusted model and 1.42 (95% CI: 1.07–1.88) for the adjusted model, with a clear linear trend across tertiles. Additional adjustment in the model for receipt of hormone, radiation and chemotherapy had no discernible effect on the relative risk estimates for overall mortality.

**Table 5:** Hazard ratios and 95% confidence intervals for overall mortality, according to subtype admixture of Luminal A breast cancers in the METABRIC cohort

	Unadjusted		Adjusted^a^	
	HR	95% CI	HR	95% CI
Median Distance				
Pure	1.00	–	1.00	–
Neither	1.48	1.10–2.00	1.12	0.83–1.53
Admixed	1.93	1.42–2.63	1.47	1.07–2.02
Distance Ratio				
Tertile 1	1.00	–	1.00	–
Tertile 2	1.45	1.11–1.91	1.20	0.91–1.59
Tertile 3	1.98	1.52–2.58	1.42	1.07–1.88
	*P*_trend_ = < 0.001		*P*_trend_ = < 0.001	

^a^Adjusted for age, grade, stage and tumor size

### Validation on TCGA cohort

**Fig. 5:**
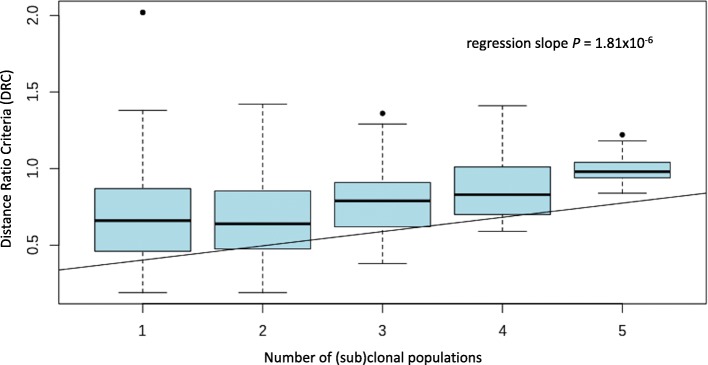
Relationship between Distance Ratio Criteria (DRC) metric for subtype admixture and number of clonal populations estimated by PyClone: Luminal A breast cancers in TCGA (*n* = 509). Box = inter-quartile range, horizontal bar = median, upper/lower whisker ~ 1.5 IQR/sqrt(n)

For independent validation of subtype admixture quantification, we used PAM50 gene expression profiles of 1081 breast cancers cases from the TCGA cohort. Additional file 2: Figure S1 illustrates the t-SNE plot of PAM50 gene expression for all TCGA cases, by assigned subtype. The 509 Luminal A cases were further stratified according to subtype purity by MDC and DRC. Clustering of the T1 through T3 subgroups in t-SNE plots shows progressive migration of Luminal A cases towards alternate subtypes (Additional file 3: Figure S2). Contrasts in clinical and molecular features across admixture groups in TCGA are similar to those observed in METABRIC (Additional file 4: Tables S2, Additional file 5: Table S3). In TCGA there was a strong positive association between DRC and the number of clonal populations estimated by PyClone; *P* = 1.81 × 10^− 6^ for a test of trend by linear regression (Fig. 5). Cases with the highest possible number of clonal populations [5] were found only in the T3 group, and cases predicted to have a single clone comprised 74.4, 63.7 and 56.6% in subgroups T1 through T3, respectively (Additional file 5: Table S3).

**Fig. 6:**
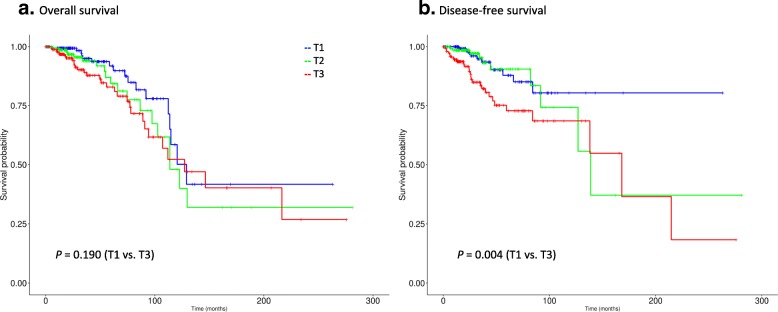
In the TCGA validation cohort, disease-free survival among Luminal A cases was significantly worse for admixed cases (**b**), as determined by Distance Ratio criteria. A similar trend is shown for overall survival (**a**), but statistical power was limited

Figure 6 shows the Kaplan-Meier plots for OS and disease-free survival (DFS) for TCGA Luminal A patients stratified by DRC. The T3 vs. T1 difference for overall survival was similar in magnitude to METABRIC although not statistically significant (Fig. 6a); however, this was most likely due to the limited number of deaths in the cohort as there was a clear difference in disease-free survival (Fig. 6b; *P* = .004). In proportional hazards models on the TCGA cohort, the risk of recurrence and overall mortality was higher in T3 cases, with evident trends across tertiles (Table 6). In multivariable models, risk of recurrence was nearly three-fold higher in T3 versus T1 (HR = 2.85). In summary, the relationships between the admixture metrics and survival in TCGA were similar to those we observed in METABRIC cohort.

**Table 6:** Hazard ratios and 95% confidence intervals for disease-free survival and overall survival, according to subtype admixture of Luminal A breast cancers in the TCGA validation cohort, Distance Ratio criteria only

	Unadjusted		Adjusted^a^	
	HR	95% CI	HR	95% CI
Disease-free survival				
Tertile 1	1.00	–	1.00	–
Tertile 2	1.19	0.51–2.81	1.14	0.48–2.74
Tertile 3	2.73	1.35–5.48	2.85	1.39–5.85
	*P*_trend_ = 0.003		*P*_trend_ = 0.002	
Overall survival				
Tertile 1	1.00	–	1.00	–
Tertile 2	1.45	0.75–2.79	1.34	0.69–2.62
Tertile 3	1.73	0.95–3.14	1.90	1.02–3.54
	*P*_trend_ = 0.074		*P*_trend_ = 0.040	

^a^Adjusted for age, stage and tumor size

## Discussion

Among the major breast cancer subtypes, Luminal A tumors are reported to have the highest inter-tumor molecular diversity, despite a lower mutation rate, as well as the highest diversity of clinical outcome [12]. However, the intra-tumor heterogeneity of these cancers has received less attention, notwithstanding evidence that they also have the highest within-tumor variance in transcriptional profile among the major intrinsic subtypes [31]. Here we postulate that detection of intra-tumor heterogeneity is at least partly a function of the mixed cell composition obtained by bulk sampling, and that ambiguity in relation to an assigned subtype can be due to admixture of multiple subtypes in an individual case. We found that stratification of Luminal A breast cancers according to the degree of subtype purity using PAM50 gene expression supports the hypothesis that admixed cases, compared to more pure ones, have worse clinical features and overall survival. The robustness of this conclusion is indicated by similar results using two different purity metrics and independent validation in an external cohort. The admixture patterns we observed suggest that Luminal A is predominantly admixed with Luminal B, as expected, although evidence for other types of admixture was also found. Our data indicate that a distance ratio metric - a continuous measure that captures the distance in multidimensional space of an individual case to its assigned centroid relative to its distance from the nearest neighboring unassigned centroid - is superior to a categorical metric that strictly divides cases into pure and admixed subgroups.

Previous studies have characterized intra-tumor heterogeneity and its significance in breast cancer in a number of more or less direct ways. In addition to the association between outcome and percentage of ER-positive cells mentioned above, the concentration of ER per tumor mass by ligand-binding assay is positively associated with response to adjuvant tamoxifen [32]. Intra-tumor heterogeneity is also implied by the ASCO/CAP criterion for a tumor to be considered HER2-positive, which requires only 10% of tumor cells with complete and intense membrane staining, and by the relationship between the heterogeneity of HER2 staining and clinical outcomes [33, 34]. Many breast cancers have discordant subtype assignments by IHC versus RNA profiling and such cases have worse features and outcomes than cases with concordant classification [35–37]. Intra-tumor heterogeneity has been measured more directly as a function of the variant allele frequency in tumor DNA using the mutant-allele tumor heterogeneity (MATH) score, and the ER+ cancers with greater heterogeneity were shown to have worse breast cancer-specific survival [38]. Multiregion targeted gene sequencing has directly demonstrated subclonal heterogeneity in all four major breast cancer subtypes, to a varying degree among patients [39]. Such diversity cannot be revealed easily by bulk sampling, which only provides an average of the subpopulation genomic profiles and tends by its nature to underestimate overall diversity [10]. Finally, whole genome analyses comparing paired primary and metastatic tissues also confirm that many breast cancers exist as mosaics that include minority subpopulations of cells [40].

The predominant admixture we observed, between Luminal A and B, was anticipated based on previous observations that a distinct boundary between these subtypes, in molecular terms, might not exist. The frequency of Luminal A confusion with Luminal B was reflected in the non-significant differences between pure and admixed cases in ER and PR positivity by IHC. Clinically, discrimination between these subtypes is often determined by measuring proliferation using IHC for Ki67, and genomic comparisons of Luminal A versus Luminal B have shown that this distinction is basically a continuum, with proliferation and cell cycle gene activity driving the tendency towards one subtype or the other [41, 42]. Assuming that the major subtypes represent favored pathways along which breast cancers can develop, multiple subtypes could logically coexist within a single tumor due to differences in cells of origin or differences in subclonal evolution, driven by heterogeneity in the tumor microenvironment over time and space [43].

Although Luminal A confusion with Luminal B was the most common finding, we also observed other types of admixture. Among the 41% of admixed cases by MDC that were not admixed with Luminal B, 23% were admixed with Claudin-low, 17% were admixed with HER2, and 1% were admixed with Basal in the METABRIC cohort. For the 35% of T3 DRC cases not linked to Luminal B, 10, 24 and 1% were admixed with Claudin-low, HER2, and Basal, respectively. Compared to more pure cases, admixed cases had a higher frequency of HER2 amplification and higher HER2 positivity by IHC or FISH in both METABRIC and TCGA, which confirms previous reports of HER2 features within the Luminal A group [12]. We also found a strong relationship between admixture and TP53 mutation, which is generally associated with non-luminal breast cancer [44]. Finally, although six of the top ten genes differentiating T3 cases from T1 were related to proliferation and cell cycle control, only one (*KIF2C*) was included in the PAM50 based mainly on its usefulness for distinguishing the Luminal B subtype, while among the other nine genes in the top ten, three were selected based on association with the Normal-like profile, two with Basal and one with HER2 (see Additional file 1: Table S1) [21].

Interestingly, the significance of subtype ambiguity and potential admixture is embedded in the PAM50-based Risk of Recurrence (ROR) score, which was initially developed using qPCR and is now validated and available for clinical use on the Nanostring platform [21, 45]. A major component in the Cox models underlying these RORs are weights derived from the Pearson correlations of an individual case to each of the four major subtype centroids. The utility of these weights as predictors indicates that the closer a single case adheres to a particular subtype paradigm, the more its predicted survival resembles that of the entire class. Thus, it is not surprising that we found a strong association between the PAM50 ROR score and our admixture metrics, which can incorporate the variance-covariance information between competing centroids.

We recognize that this study has both strengths and limitations. Its strengths include analysis of a large number of Luminal A cancers, and validation supported by similar results involving two different metrics and testing in an independent cohort. However, we had limited statistical power for evaluating survival endpoints for admixture of Luminal A with other specific subtypes. In addition, evaluating only 50 genes could have limited sensitivity for measuring admixture. Expression levels for three genes were not available in the METABRIC dataset, however, their inclusion would be likely to either increase or have no effect on the trends we observed. Moreover, three PAM50 genes were also excluded from previous METABRIC publications and the Prosigna™ PAM50 assay excluded four genes with no apparent effect on classification error or prognostic accuracy [45].

We note that, although we found similar results in METABRIC and TCGA, caution is always indicated when generalizing results from one historical cohort to another, since differences in patient populations and changes in treatment patterns can influence associations with tumor characteristics and survival. With regard to treatment, we note that in the METABRIC cohort a large proportion of ER-positive Luminal A patients (32%) did not receive hormone therapy, and no patients with HER2-positive cancers received anti-HER2 therapy, indicating the need to study cohorts with more contemporary treatment experience. Contamination of the bulk tumor samples with either stromal or benign breast cells is a concern. However, none of the PAM50 genes are highly expressed in stroma and thus are unlikely to have distorted expression patterns. Moreover, benign epithelilal cells are expected to be few in number relative to tumor cells. In either case, the degree of such contamination should be unrelated to true PAM50 purity and thus would conservatively bias any of the observed associations towards the null.

The absence of an association between admixture among Luminal A cases and mutational load or MATH score is somewhat puzzling and warrants further exploration. The sensitivity of the MATH score for indicating heterogeneity in TCGA was questionable because only 173 putative driver genes underwent whole exome sequencing. However, in METABRIC the null association could be due to counteracting forces since, overall, Luminal A tumors tend to have fewer but more diverse sets of mutations than other subtypes [44]. Thus, admixed Luminal A cases could lose some intra-tumor diversity compared to pure ones but also gain some due to the presence of multiple subtypes with different mutation patterns in the same tumor. In contrast to the null association for mutational load and MATH score, we found that the number of clonal populations estimated by PyClone was strongly associated with subtype admixture. PyClone provides a more direct indication of the clonal structure of the tumor while also controlling for the effect of variation in copy number. We observed minimal correlation between clone number and MATH score or mutational load in our data, an observation also made by Morris, et al. in a pan-cancer analysis [16].

Our finding that admixed cases tend to be older at diagnosis is also somewhat counterintuitive, since Luminal A patients are generally older than patients in the other major subtypes [46]. However, among breast cancers of all types in a large cohort, older age was associated with higher Ki67 expression, despite the older age of Luminal A patients [47]. One possible explanation is that, within the Luminal A group, it takes longer in admixed cases for subclones resembling more aggressive alternate subtypes to evolve and expand. This hypothesis is consistent with results from a pan-cancer analysis, which found that the number of SNVs in large clones was positively correlated with age at diagnosis [14].

Future directions for research could include combining cohorts for greater power to assess specific types of admixture and exploiting methods to use whole transcriptome data and integrative genomics to measure admixture with greater accuracy. In addition, the admixture metrics developed here could be studied in paired primary-metastatic tissue samples, to help clarify the role of intrinsic heterogeneity in tumor progression and treatment response. Incorporation of copy number data into admixture analysis would be particularly interesting. Ciriello, et al. used copy number data in METABRIC and TCGA to identify distinct subclasses within the Luminal A group [12]. The most aggressive subclass found, called Copy Number High, had several characteristics in common with our most admixed cases, including high proliferation, high prevalence of *TP53* mutation, and low prevalence of *PIK3CA* mutation. Finally, we recognize that the admixture metrics we developed are derived from bulk samples and thus are subject to undersampling and furthermore require the supposition that ambiguous gene profiles correspond to spatial diversity within a tumor. Therefore, future studies will focus on incorporating spatial information to provide definitive evidence for this premise [13, 48–50].

## Conclusion

We have developed and tested intuitive metrics indicative of subtype admixture based on PAM50 genes in bulk sampled tissue, and demonstrated that the degree of adherence of a Luminal A breast cancer to its class prototype is associated with its clinical features and survival. These findings call attention to the importance of the potential relationship between subtype ambiguity and intra-tumor heterogeneity as represented by the coexistence of multiple subtypes in a single tumor.

## Additional files

Additional file 1: **Table S1.** Top ten genes ranked by differences in gene expression between Tertile 1 (pure) and Tertile 3 (admixed) Luminal A cases in METABRIC. (DOCX 17 kb)

Additional file 2: **Figure S1.** t-SNE plots for all subtypes in the TCGA cohort, showing: 1) luminal subtypes cluster relatively closely, with Basal cluster highly distant, 2) substantial admixture across subtypes as indicated by cases located near non-assigned centroids. (centroids = open circles) (PPTX 2616 kb)

Additional file 3: **Figure S2.** t-SNE plots showing subtype clustering of Luminal A cases in the TCGA cohort according to degree of admixture based on Distance Ratio tertile. Luminal A cases progressively migrate towards other subtype regions, while predominantly co-clustering with Luminal B. The pattern is similar to that observed in METABRIC. (PPTX 93 kb)

Additional file 4: **Table S2.** Clinical characteristics of patients in the TCGA cohort with Luminal A breast cancer, classified by Distance Ratio purity. (DOCX 20 kb)

Additional file 5: **Table S3.** Molecular characteristics of Luminal A breast cancers in the TCGA cohort classified by subtype purity. (DOCX 24 kb)

## Abbreviations

ASCO/CAP: American Society for Clinical Oncology/College of American Pathologists
DRC: Distance Ratio Criteria
IHC: immunohistochemistry
MATH: Mutant Allele Tumor Heterogeneity
MDC: Median Distance Criteria
METABRIC: Molecular Taxonomy of Breast Cancer International Consortium
PAM50: Prediction Analysis of Microarray 50
ROR: Risk of Recurrence
TCGA: The Cancer Genome Atlas
t-SNE: t-distributed Stochastic Neighbor Embedding
