# Supplementary material for: Quantification of intrinsic subtype ambiguity in Luminal A breast cancer and its relationship to clinical outcomes
Source: BMC Cancer. 2019 Mar 8;19:215. doi: 10.1186/s12885-019-5392-z (PMC6408846; doi:10.1186/s12885-019-5392-z)
Supplement: Supplementary file 1 — Table S1. Top ten genes ranked by differences in gene expression between Tertile 1 (pure) and Tertile 3 (admixed) Luminal A cases in METABRIC. (DOCX 17 kb) [file 12885_2019_5392_MOESM1_ESM.docx]

| **Table S1.** Top ten genes ranked by differences in gene expression between Tertile 1 (pure) and Tertile 3 (admixed) Luminal A cases in METABRIC | | | | | | | |
| --- | --- | --- | --- | --- | --- | --- | --- |
| Gene | Mean mRNA expression^a^ | | | *P* value^b^ | Proliferation? | Subtype annotation^c^ | In LumA centroid^d^ |
|  | T1 | T2 | T3 |  |  |  |  |
| *UBE2C* | 9.237 | 9.352 | 9.427 | 2.87E-45 | Yes | LumA | Down |
| *CEP55* | 8.965 | 9.032 | 9.074 | 3.95E-41 | Yes | Basal | Down |
| *PTTG1* | 9.284 | 9.367 | 9.427 | 1.47E-38 | Yes | LumA | Down |
| *CDC20* | 9.229 | 9.311 | 9.384 | 1.64E-35 | Yes | HER2 | Down |
| *MELK* | 9.001 | 9.076 | 9.130 | 7.61E-33 | No | Basal | Down |
| *KIF2C* | 8.896 | 8.953 | 8.998 | 1.56E-32 | No | LumB | Down |
| *KRT14* | 9.217 | 9.059 | 8.967 | 1.62E-32 | No | Normal | Up |
| *BIRC5* | 8.926 | 9.000 | 9.056 | 2.22E-32 | Yes | LumA | Down |
| *KRT5* | 9.018 | 8.908 | 8.837 | 1.31E-31 | No | Normal | Up |
| *CCNB1* | 8.898 | 8.954 | 8.997 | 4.41E-29 | Yes | Normal | Down |
| ^a^ Log-transformed, normalized, and scaled gene expression values  ^b^ *P* value for T1 vs. T3 by t test  ^c^ PAM50 subtype most strongly differentiated by each gene  ^d^ Direction of expression in LumA centroid relative to other subtypes | | | | | | | |
