# Supplementary material for: Quantification of intrinsic subtype ambiguity in Luminal A breast cancer and its relationship to clinical outcomes
Source: BMC Cancer. 2019 Mar 8;19:215. doi: 10.1186/s12885-019-5392-z (PMC6408846; doi:10.1186/s12885-019-5392-z)
Supplement: Supplementary file 2 — Figure S1. t-SNE plots for all subtypes in the TCGA cohort, showing: 1) luminal subtypes cluster relatively closely, with Basal cluster highly distant, 2) substantial admixture across subtypes as indicated by cases located near non-assigned centroids. (centroids = open circles) (PPTX 2616 kb) [file 12885_2019_5392_MOESM2_ESM.pptx]

## Slide 1
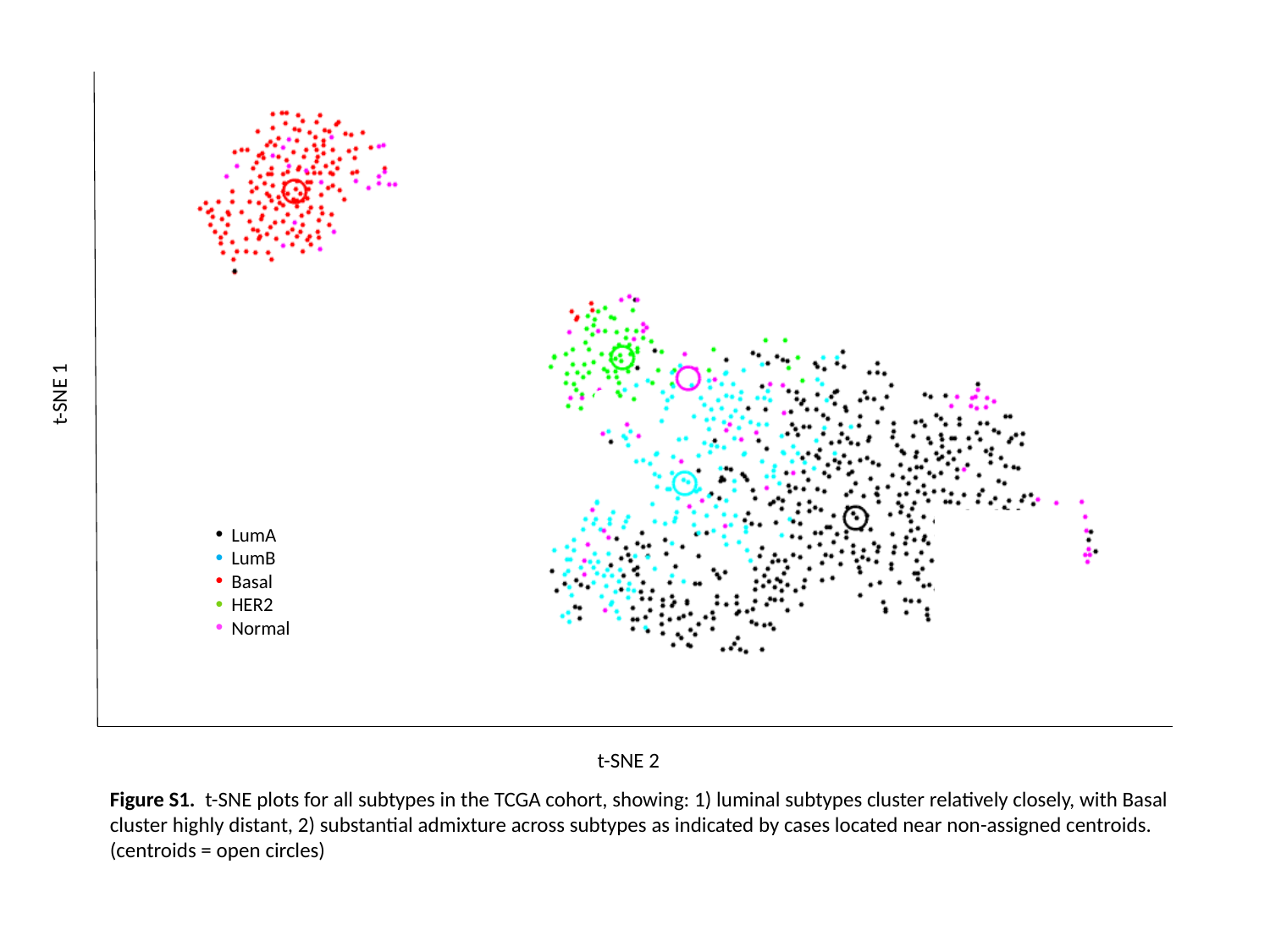

t-SNE 1
t-SNE 2
LumA
LumB
Basal
HER2
Normal
Figure S1. t-SNE plots for all subtypes in the TCGA cohort, showing: 1) luminal subtypes cluster relatively closely, with Basal cluster highly distant, 2) substantial admixture across subtypes as indicated by cases located near non-assigned centroids. (centroids = open circles)
