# Supplementary material for: Quantification of intrinsic subtype ambiguity in Luminal A breast cancer and its relationship to clinical outcomes
Source: BMC Cancer. 2019 Mar 8;19:215. doi: 10.1186/s12885-019-5392-z (PMC6408846; doi:10.1186/s12885-019-5392-z)
Supplement: Supplementary file 3 — Figure S2. t-SNE plots showing subtype clustering of Luminal A cases in the TCGA cohort according to degree of admixture based on Distance Ratio tertile. Luminal A cases progressively migrate towards other subtype regions, while predominantly co-clustering with Luminal B. The pattern is similar to that observed in METABRIC. (PPTX 93 kb) [file 12885_2019_5392_MOESM3_ESM.pptx]

## Slide 1
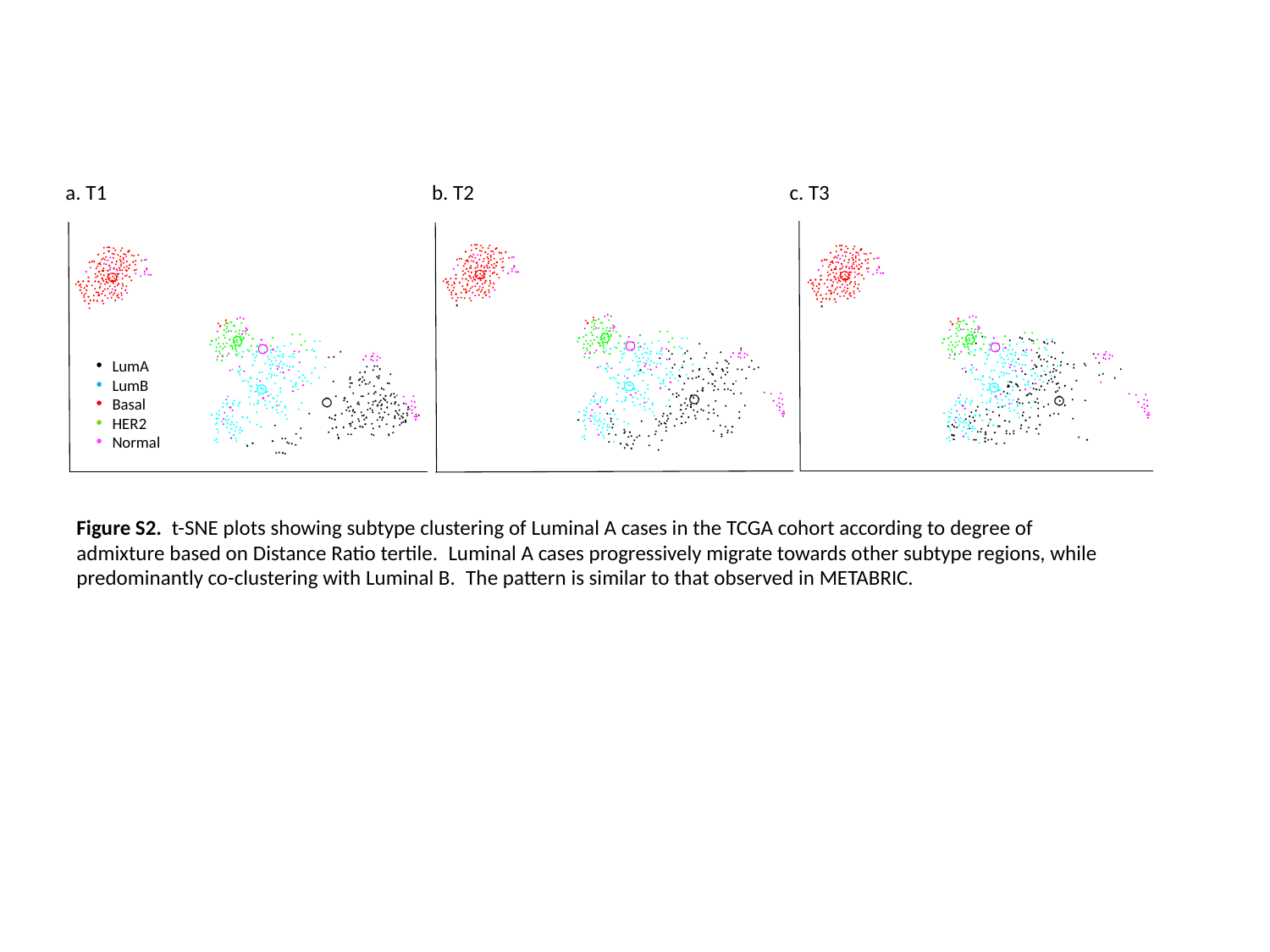

c. T3
a. T1
b. T2
LumA
LumB
Basal
HER2
Normal
Figure S2. t-SNE plots showing subtype clustering of Luminal A cases in the TCGA cohort according to degree of admixture based on Distance Ratio tertile.  Luminal A cases progressively migrate towards other subtype regions, while predominantly co-clustering with Luminal B.  The pattern is similar to that observed in METABRIC.
