# Supplementary material for: Quantification of intrinsic subtype ambiguity in Luminal A breast cancer and its relationship to clinical outcomes
Source: BMC Cancer. 2019 Mar 8;19:215. doi: 10.1186/s12885-019-5392-z (PMC6408846; doi:10.1186/s12885-019-5392-z)
Supplement: Supplementary file 4 — Table S2. Clinical characteristics of patients in the TCGA cohort with Luminal A breast cancer, classified by Distance Ratio purity. (DOCX 20 kb) [file 12885_2019_5392_MOESM4_ESM.docx]

| **Table S3.** Molecular characteristics of Luminal A breast cancers in the TCGA cohort classified by subtype purity | | | | | | | | |
| --- | --- | --- | --- | --- | --- | --- | --- | --- |
|  | Median distance criteria | | | | Distance ratio tertiles | | | |
|  | Pure  n = 125 | Neither  n = 338 | Admixed  n = 46 | *P*,  Pure vs Admixed | T1  n = 168 | T2  n = 168 | T3  n = 173 | *P*,  T1 vs T3 |
| Proliferation Score, PAM50 (mean) | 7.90 | 8.21 | 8.80 | <0.001 | 7.50 | 8.33 | 8.74 | <0.001 |
| Recurrence Score, PAM50 (mean) | 17.44 | 34.16 | 38.33 | <0.001 | 14.19 | 32.24 | 44.44 | <0.001 |
| Risk of recurrence, PAM50  Low  Intermediate  High | 85%  14%  1% | 36%  53%  11% | 19%  70%  11% | <0.001 | 92%  8%  0% | 42%  54%  4% | 7%  71%  22% | <0.001 |
| Mutational load^a^ (mean) | 42.37 | 64.31 | 37.48 | 0.798 | 50.14 | 73.23 | 46.26 | 0.812 |
| MATH score^b^ (mean) | 0.36 | 0.38 | 0.36 | 0.992 | 0.37 | 0.36 | 0.39 | 0.181 |
| No. of clonal populations, n (%)^c^ |  |  |  |  |  |  |  |  |
| 1 | - | - | - | - | 125 (74.4) | 107 (63.7) | 98 (56.6) | <0.001 |
| 2 | - | - | - | - | 34 (20.2) | 26 (15.5) | 26 (15.0) | - |
| 3 | - | - | - | - | 9  (5.4) | 21  (12.5) | 20  (11.6) | - |
| 4 | - | - | - | - | 0  (0.0) | 14  (8.3) | 16  (9.3) | - |
| 5 | - | - | - | - | 0  (0.0) | 0  (0.0) | 13  (7.5) | <0.001 |
| Somatic mutations (%) |  |  |  |  |  |  |  |  |
| TP53 | 7% | 12% | 9% | 0.998 | 3% | 12% | 16% | <0.001 |
| PIK3CA | 58% | 34% | 41% | 0.069 | 51% | 41% | 29% | <0.001 |
| CBFB | 4% | 3% | 9% | 0.404 | 4% | 2% | 5% | 0.312 |

^a^ Number of mutations among 11610 selected genes sequenced.

^b^ MATH score = mad(vaf)/median(vaf); where mad=median absolute deviation and vaf=variant allele frequency

(intratumor heterogeneity based on variation in mutant allele frequency, see Mroz EA, Oral Oncol 2013)

^c^ PyClone estimated number of clonal populations in tumor (see Roth A, Nat Methods 2014)
