# Supplementary material for: Quantification of intrinsic subtype ambiguity in Luminal A breast cancer and its relationship to clinical outcomes
Source: BMC Cancer. 2019 Mar 8;19:215. doi: 10.1186/s12885-019-5392-z (PMC6408846; doi:10.1186/s12885-019-5392-z)
Supplement: Supplementary file 5 — Table S3. Molecular characteristics of Luminal A breast cancers in the TCGA cohort classified by subtype purity. (DOCX 24 kb) [file 12885_2019_5392_MOESM5_ESM.docx]

| **Table S2.** Clinical characteristics of patients in the TCGA cohort with Luminal A breast cancer, classified by Distance Ratio Criteria (DRC) purity | | | | |
| --- | --- | --- | --- | --- |
|  | Distance ratio tertile | | |  |
|  | T1  n = 168 | T2  n = 168 | T3  n = 173 | *P*,  T1 vs T3 |
| Age, years (mean) | 58.3 | 60.3 | 59.3 | 0.486 |
| Menopausal status (%) |  |  |  |  |
| Pre-menopausal | 23% | 21% | 20% | 0.737 |
| Post-menopausal | 65% | 63% | 64% | - |
| Indeterminate | 12% | 17% | 16% | - |
| ER+ (%) | 95% | 96% | 91% | 0.901 |
| PR+ (%) | 90% | 91% | 78% | 0.476 |
| HER2+ (%) | 8% | 9% | 17% | 0.005 |
| Tumor size (%) |  |  |  |  |
| < 20 mm | 36% | 35% | 24% | 0.031 |
| > 20 mm | 64% | 65% | 75% | - |
| Node positive (%)  Missing | 45%  6% | 45%  13% | 47%  17% | 0.115 |
| Stage (%)^a^ |  |  |  |  |
| I | 23% | 25% | 14% | 0.066 |
| II-III-IV | 76% | 71% | 82% | - |

^a^  Excluding missing data (2-3%).
